# Structure–function correlates of fibrinogen binding by *Acinetobacter* adhesins critical in catheter-associated urinary tract infections

**DOI:** 10.1073/pnas.2212694120

**Published:** 2023-01-18

**Authors:** Kevin O. Tamadonfar, Gisela Di Venanzio, Jerome S. Pinkner, Karen W. Dodson, Vasilios Kalas, Maxwell I. Zimmerman, Jesus Bazan Villicana, Gregory R. Bowman, Mario F. Feldman, Scott J. Hultgren

**Affiliations:** ^a^Department of Molecular Microbiology, Washington University School of Medicine, St Louis, MO 63110; ^b^Center for Women’s Infectious Disease Research, Washington University School of Medicine, St Louis, MO 63110; ^c^Department of Medicine, McGaw Medical Center of Northwestern University, Chicago, IL 60611; ^d^Department of Pathology and Immunology, Washington University School of Medicine, St. Louis, MO 63110; ^e^Department of Biochemistry & Molecular Biophysics, Washington University School of Medicine, St. Louis, MO 63110; ^f^Department of Biomedical Engineering and Center for Biological Systems Engineering, Washington University School of Medicine, St. Louis, MO 63110

**Keywords:** *Acinetobacter baumannii*, catheter-associated urinary tract infections, chaperone-usher pathway pili, adhesins

## Abstract

Multidrug-resistant *Acinetobacter baumannii* infections are an urgent clinical problem and can cause difficult-to-treat nosocomial infections. During such infections, like catheter-associated urinary tract infections (CAUTI), *A. baumannii* rely on adhesive, extracellular fibers, called chaperone-usher pathway (CUP) pili for critical binding interactions. The *A. baumannii* uropathogenic strain, UPAB1, and the pan-European subclone II isolate, ACICU, use the CUP pili Abp1 and Abp2 (previously termed Cup and Prp, respectively) in tandem to establish CAUTIs, specifically to facilitate bacterial adherence and biofilm formation on the implanted catheter. Abp1 and Abp2 pili are tipped with two domain tip adhesins, Abp1D and Abp2D, respectively. We discovered that both adhesins bind fibrinogen, a critical host wound response protein that is released into the bladder upon catheterization and is subsequently deposited on the catheter. The crystal structures of the Abp1D and Abp2D receptor-binding domains were determined and revealed that they both contain a large, distally oriented pocket, which mediates binding to fibrinogen and other glycoproteins. Genetic, biochemical, and biophysical studies revealed that interactions with host proteins are governed by several critical residues in and along the edge of the binding pocket, one of which regulates the structural stability of an anterior loop motif. K34, located outside of the pocket but interacting with the anterior loop, also regulates the binding affinity of the protein. This study illuminates the mechanistic basis of the critical fibrinogen-coated catheter colonization step in *A. baumannii* CAUTI pathogenesis.

## Significance

Multidrug-resistant *Acinetobacter baumannii* is a major public health threat, causing difficult-to-treat nosocomial infections. We identified two virulence pili independently critical to *A. baumannii* catheter-associated urinary tract infections, the chaperone-usher pathway pili Abp1 and Abp2. Mechanistically, these pili contribute to *A. baumannii* biofilm formation. Further, their respective tip-adhesive proteins, the adhesins Abp1D and Abp2D, make use of large, distally oriented binding pockets to adhere to glycoproteins like fibrinogen, a wound-healing protein important in CAUTI pathogenesis.

*Acinetobacter baumannii* is a gram-negative, opportunistic pathogen able to colonize a variety of human host tissues (1). Among the most common infections caused by *A. baumannii* are urinary tract, bloodstream, indwelling catheter, and respiratory infections (1, 2). In addition to its opportunistic versatility, antibiotic and multidrug resistance is a significant concern for *A. baumannii.* The World Health Organization and Centers for Disease Control and Prevention have designated it an ‘urgent’ threat and a ‘critical’ microorganism target for the development of new antibiotics (3). A largely underappreciated clinical problem posed by *A. baumannii* is its ability to establish catheter-associated urinary tract infections (CAUTI). Catheterization is a common medical procedure in a hospital setting, wherein tubing is inserted into the bladder lumen to allow for the voiding of the bladder (4). This process can result in the wounding of the bladder and the release of fibrinogen into the bladder lumen, which subsequently coats the catheter (5). Interestingly, uropathogens have evolved fibrinogen-binding adhesins that mediate binding and biofilm formation on the catheter leading to infection in a mouse model of CAUTI (6, 7), and ultimately, such infections are a major driver of nosocomial infections (4, 8). A recent study developed a murine model of *A. baumannii* CAUTI and demonstrated bacterial colonization of both the bladder and catheter by the clinical isolate UPAB1 (1). That study found that a double mutant strain that deletes two chaperone usher pathway (CUP) pilus operons, UPAB1(Δ*abp1*Δ*abp2*), is attenuated in CAUTI (9). Abp1 and Abp2 pili were previously referred to as CUP1 (*prpA-D*) and CUP2 (*cupA-D*), respectively. These pili belong to the γ4 clade of the CUP pilus family, of which over 100 members have been identified in Gram-negative bacteria (10, 11). CUP pili are a family of pili which are assembled by a common molecular machinery that catalyzes pilus biogenesis using respective periplasmic chaperones and outer membrane ushers (11, 12). The periplasmic chaperone is composed of two complete Ig domains arranged in a boomerang shape, which transiently completes the Ig fold of subunits in the periplasm to catalyze subunit folding via donor strand complementation (12). The usher is a five-domain outer-membrane protein (13, 14) which catalyzes the assembly of individual pilus subunits together via donor strand exchange (DSE) and secretion of the pilus fiber (13). In addition to encoding a periplasmic chaperone and usher, each CUP gene operon can encode: i) a major subunit, which polymerizes to form the homopolymeric pilus rod (12); and ii) an adhesin, a two-domain protein that is located on the tip of the final pilus fiber with an N-terminal receptor-binding (or lectin) domain, and a C-terminal pilin domain that anchors the adhesin to the pilus shaft (10, 11, 14, 15). Each pilin subunit is an incomplete Ig fold missing a C-terminal beta strand. Every subunit also has an N-terminal extension that completes the Ig fold of its neighboring subunit in the pilus via a DSE reaction catalyzed by the usher (16).

CUP pilus adhesins recognize specific receptors to mediate host–pathogen interactions critical in bacterial pathogenesis (17–19). The *A. baumannii* Abp1 and Abp2 pili are tipped with the Abp1D and Abp2D adhesins, respectively. We found that the deletion of each pilus gene cluster was sufficient to attenuate uropathogenesis in a CAUTI mouse model (1). Thus, we investigated the structure–function correlates of the Abp1D and Abp2D adhesins by crystallizing and solving the structures of the receptor-binding domains (RBDs) of each adhesin. We demonstrate that Abp1D and Abp2D RBDs are structurally similar, and each binds fibrinogen and other relevant host glycoproteins important in CAUTI and other infections. These proteins share a common binding mechanism, using a large, distally oriented, and flexible pocket that recognizes a complex glycan receptor. Binding is also controlled by a key residue outside of the pocket which makes intramolecular interactions with the pocket that regulates protein function. This work contributes to our knowledge of the *A. baumannii* mechanism of CUP pilus-dependent CAUTI.

## Results

### Abp1 and Abp2 Pili Are Present in the Majority of *A. baumannii*.

To determine the prevalence of Abp1 and Abp2 pili in isolates of *A. baumannii*, we surveyed the European Nucleotide Archive (20) for the presence of the adhesins *abp1D* and *abp2D*. We identified 5,201 unique *A. baumannii* genomes with 44% (2,267) possessing *abp1D* and 51% (2,642) possessing *abp2D*. 31% (1,630) of the *A. baumannii* genomes possessed both *abp1D* and *abp2D*. Only 37% (1,922) of 5,201unique genomes contain neither *abp1D* nor *abp2D*. Thus, these genes are prevalent and co-occur frequently. Among Multidrug-resistant (MDR) isolates containing both pili are the uropathogen UPAB1 (1) and the cerebrospinal fluid isolate ACICU, a member of the international clone 2, which together with IC1, are responsible for most MDR *Acinetobacter* outbreaks (21).

### Abp1 and Abp2 Pili Mediate Critical Host–Pathogen Interactions in *A. baumannii* CAUTI.

**Fig. 1.**
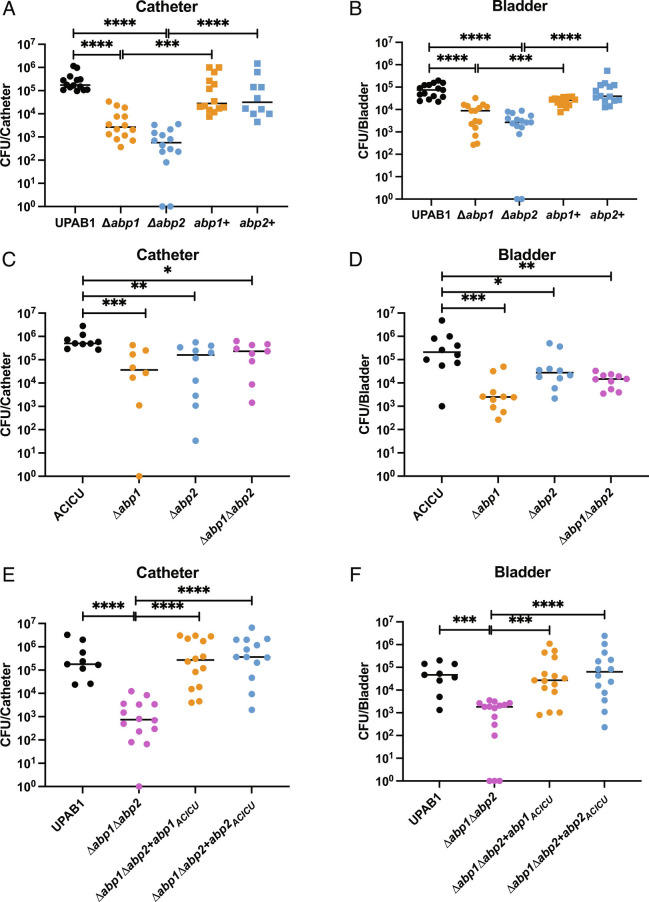
*A. baumannii* CAUTI is CUP pilus mediated. BL6 mice infected with 10^8^ cells of UPAB1 or ACICU in a CAUTI model with catheter and bladder harvested and processed for CFUs at 24 hpi. (*A* and *B*) UPAB1 CAUTI model with catheter and bladder CFUs quantified, respectively. (*C* and *D*) ACICU CAUTI model with catheter and bladder CFUs quantified, respectively. (*E* and *F*) UPAB1 CAUTI model with CUP pilus mutants complemented with pilus operons from ACICU; (*E*) catheter and (*F*) bladder CFUs quantified. Mann-Whitney test. ****P ≤ 0.01, ***P ≤ 0.01, **P ≤ 0.01, *P ≤ 0.05.

Previous work has shown that a double deletion mutant UPAB1(Δ*abp1*Δ*abp2*) strain is attenuated in a mouse CAUTI model (1). To assess the individual contribution of each pilus to uropathogenesis, we deleted each pilus gene cluster individually and tested the ability of the respective mutant strains to cause CAUTI in C57BL/6 mice. Catheter and bladder colonization decreased for each single CUP pili mutant of UPAB1 (UPAB1Δ*abp1* and UPAB1Δ*abp2*) compared to WT (Fig. 1 *A* and *B*). Likewise, CAUTI by the ACICU strain is dependent on both Abp1 and Abp2 pili in vivo, with each pilus deletion (ACICUΔ*abp1* and ACICUΔ*abp2*) being attenuated in colonizing the catheter and bladder in the CAUTI model (Fig. 1 *C* and *D*). Complementing the UPAB1 double-deletion mutant strain (UPAB1Δ*abp1*Δ*abp2*) with either *abp1*_ACICU_ or *abp2*_ACICU_ shows that each individual pilus is capable of fully restoring both bladder and catheter colonization (Fig. 1 *E* and *F*). This indicates that these conserved virulence factors, Abp2 and Abp1 pili, even when originating from a nonuropathogen isolate, are sufficient to mediate CAUTI bladder and catheter colonization.

**Fig. 2.**
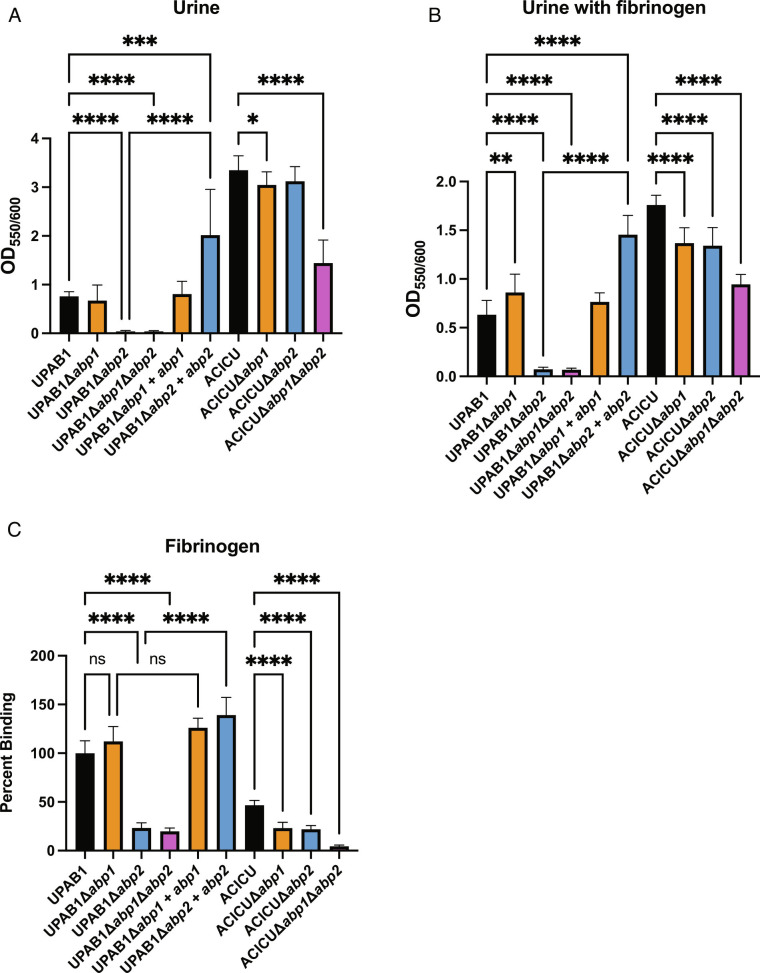
*A. baumannii* CUP pili mediate biofilm and cell adherence. Biofilm formation in *A* urine or *B* urine with 150 µg/mL fibrinogen. Cell adherence as a percentage of WT to C fibrinogen. Brown-Forsythe and Welch ANOVA tests. ****P ≤ 0.001, ***P ≤ 0.01,  **P ≤ 0.01, *P ≤ 0.05.

We found that ACICU expresses both Abp1 and Abp2 pili after growth in human urine at 37 °C (*SI Appendix*, Fig. 1*A*). Further, both ACICUΔ*abp1* and ACICUΔ*abp2* strains are deficient in binding to fibrinogen compared to WT (Fig. 2 *B* and *C*). ACICUΔ*abp1*Δ*abp2* has a significantly larger defect compared to WT in urine biofilm formation and fibrinogen binding relative to either of the single deletions (Fig. 2 *B* and *C*), suggesting that these pili are used simultaneously. Thus, in ACICU, both Abp1 and Abp2 pili seem to work in tandem to mediate the adhesive and colonization phenotypes we see *in vitro* and in vivo. In contrast to ACICU, UPAB1 expresses Abp2 pili but not Abp1 pili when grown in urine (*SI Appendix*, Fig. S1*B*). UPAB1Δ*abp2* and UPAB1Δabp1Δ*abp2* each exhibited a defect in their ability to form biofilms in vitro with and without fibrinogen at 37 °C compared to WT (Fig. 2*A*). In addition, UPAB1Δ*abp2* and UPAB1Δ*abp1*Δ*abp2* each had a significant reduction in their ability to bind fibrinogen. UPAB1Δ*abp1* is not diminished in either biofilm or binding assays (Fig. 2 *C* and *D*) compared to wild type. Therefore, while both pili were found to be critical for UPAB1 CAUTI, only Abp2 is shown to be important for biofilm formation and fibrinogen binding in vitro.

### The RBD of Abp1D and Abp2D Exhibit Similar Canonical Structures.

**Fig. 3.**
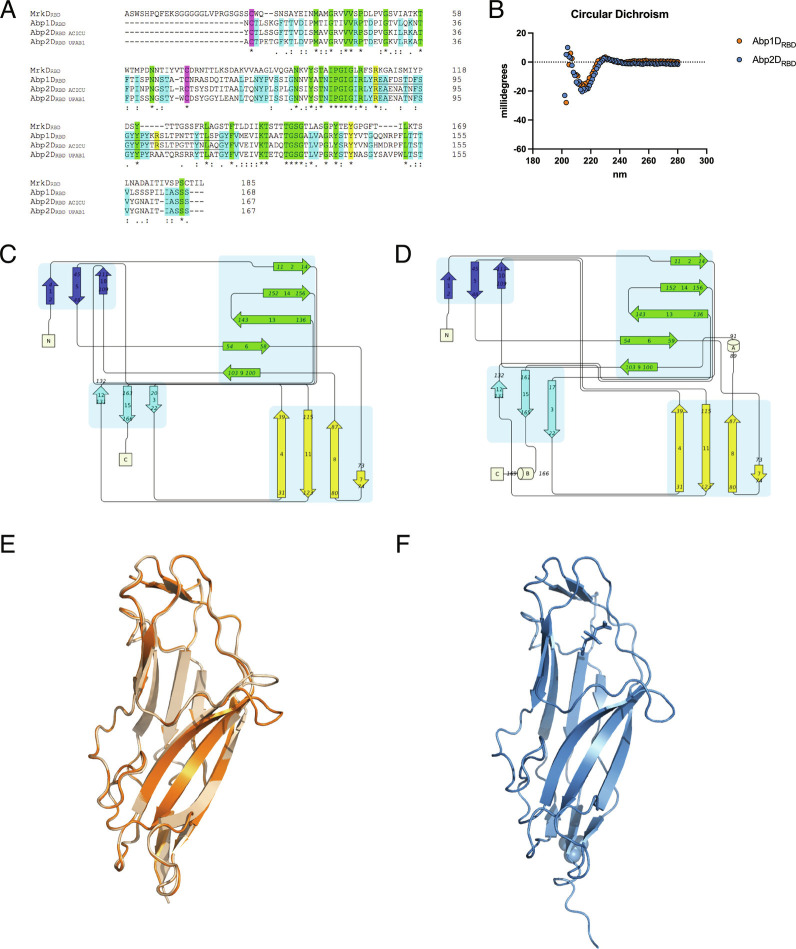
Structural features of Abp1D_RBD_ and Abp2D_RBD_. (*A*) Receptor binding domain alignment of MrkD (3U4K), Abp1D, Abp2D_ACICU_, and Abp2D_UPAB1_. Alignment by Clustal Omega. Purple—disulfide bond cysteines. Green—amino acid identical to all four amino acid sequences. Blue—residues identical to the three *Acinetobacter* sequences. Yellow—residues of interest in RBD binding mechanism. (*B*) Circular dichroism spectrum of Abp1D_RBD_ and Abp2D_RBD_ in 20 mM Phosphate 7.00. Portion of spectrum shown is as indicated by the axes here and as in the supplemental materials. (*C* and *D*) Topology maps of Abp1D_RBD_ and Abp2D_RBD_ (Pro-Origami). (*E* and *F*) Crystal structures of Abp1D_RBD_ (molecule A in orange and molecule B in wheat, rmsd 0.176 Å) (8DF0) and Abp2D_RBD_ (blue) (8DEZ) (PyMol).

The RBD of Abp1D (referred to as “Abp1D_RBD_”; identical between UPAB1 and ACICU) and Abp2D (referred to as “Abp2D_RBD_”) from ACICU were cloned, expressed, purified, and crystallized. The primary amino acid sequences of the Abp1D and Abp2D RBD are >50% identical to one another (Fig. 3*A* and *SI Appendix*, Fig. S2) (22). The structures of Abp1D_RBD_ (2.1 Å) (8DF0) (tetragonal crystals) and Abp2D_RBD_ (from ACICU) (1.3 Å) (8DEZ) (long, needle-like crystals) reveal canonical β (beta)-strand jelly-roll structures. Circular dichroism (CD) reflects the secondary structure observed in the crystallographic models for both Abp1D_RBD_ and Abp2D_RBD_ with a spectral peak consistent with β-sheets elements (Fig. 3*B*), as expected for an Ig-like protein domain. Topographically, the RBDs are organized into 4 distinct sets of β-sheets, with a single disulfide bond between C2 and C48 (noted in purple in Fig. 3*A*). In Abp2D_RBD_, two 3_10_ helices are present, one at the C-terminus and one between β strands 8 and 9. The C-terminal helix results from the six histidine tag added to this construct for purification. However, the 3_10_ helix between β strands 8 and 9 forms the anterior loop of a large, open depression (>200 Å^3^) located at the distal end of the RBD relative to the linker to the pilin domain (and by extension, pilus rod) (Fig. 3 *C*–*F* and *SI Appendix*, Fig. S3). This anterior loop motif is conserved between Abp1D_RBD_ and Abp2D_RBD._ Interestingly, other lectin domains in the γ4 clade, including MrkD (*Klebsiella* type 3 pilus, 3U4K) and GafD (*Escherichia* F17 pilus, 1OIO), contain an analogous loop. In these structures, this anterior loop contributes to the binding pocket architecture. Below, we show that the conserved loop motif also contributes to the binding of Abp1D and Abp2D, and thus, we refer to this region bound by this loop as the binding pocket.

### Structure–Function Analysis of the Abp2D and Abp1D Binding Pockets.

**Fig. 4.**
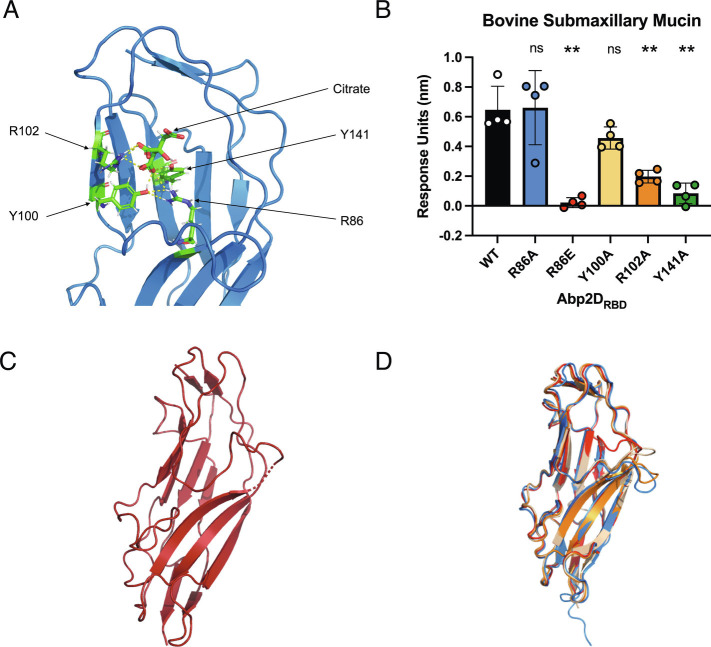
Defining the binding pocket of Abp2D_RBD_. (*A*) Citrate in the binding pocket of Abp2D_RBD_. (*B*) End time point association of biolayer inferometry assay of Abp2D_RBD_ binding pocket mutants against biotinylated BSM. (*C*) Abp2D_RBD_ R86E crystal structure (8 DKA). (*D*) Overlaid structures of the two molecules of Abp1D_RBD_ (molecule A—orange and molecule B—wheat) and Abp2D_RBD_ (blue), and Abp2D_RBD_ R86E (red). RSMD = 2.275 Å for alignment of the anterior loops of the two molecules of Abp1D_RBD_ (R86-S95) compared to 0.176 Å overall.

In the Abp2D_RBD_ crystal structure, a citrate molecule from the crystallization buffer is located within the putative binding pocket and is, in part, bound by the anterior loop (described above). The carboxylic acid residues of the citrate are coordinated by the side chains of the amino acids R86, Y100, R102, and Y141 (Fig. 4*A*). Y100 and R102 sit on the left-hand ridge of the binding site, while the base is composed of the Y141 side chain, which emanates from a β sheet on the opposite side of the receptor-binding domain core (β strand 13) and R86, situated on β strand 8 (Figs. 3*D* and 4*A*). All of the residues interacting with citrate in Abp2D_RBD_ are conserved in the Abp1D_RBD_, while all but R102 are conserved on Abp2D_RBD_ from UPAB1 (Fig. 3*A*). To test the role of these residues, their corresponding codons in *abp2D* were each mutated to alanine to encode R86A, Y100A, R102A, and Y141A variants of Abp2D_RBD_. Alanine mutations eliminated the charged, hydrophilic, polar, and aromatic side chain properties of arginine and tyrosine, respectively. The mutant proteins were purified and used to test the specific contribution of the WT side chains to adherence. We assayed their ability to bind to the glycosylated bovine submaxillary mucin (BSM) using biolayer interferometry (BLI). The R102 and Y141A mutations reduced binding relative to Abp2D_RBD_ WT by approximately ~70% and ~90%, respectively. Surprisingly, R86A Abp2D_RBD_ maintained adherence to BSM. We next inverted the side chain charge at position 86, creating R86E, to attempt to actively repel adherence. This R86E mutation abolished Abp2D binding to BSM (Fig. 4*B*). An R86E variant of Abp1D_RBD_ similarly abolished binding to BSM as measured by enzyme-linked immunosorbent assay (ELISA) (*SI Appendix*, Fig. S5 *A*–*C*). Appropriate folding of the R86E variants was demonstrated by both size exclusion chromatography (SEC) for Abp2D_RBD_ and CD for both Abp1D_RBD_ and Abp2D_RBD_ (*SI Appendix*, Fig. S4). This is also relevant to other γ4 clade adhesins. R105 in MrkD is placed in an analogous location as R86 in Abp1D and Abp2D, and R105E also eliminates MrkD_RBD_ binding (23). When aligned with GafD, whose binding pocket is known, the R86 is located in an analogous region to the pocket of GafD. This suggests that γ4 clade adhesins, as a whole, contain structurally analogous binding regions.

### Analysis of the Dynamics of Anterior Loop Movement.

**Fig. 5.**
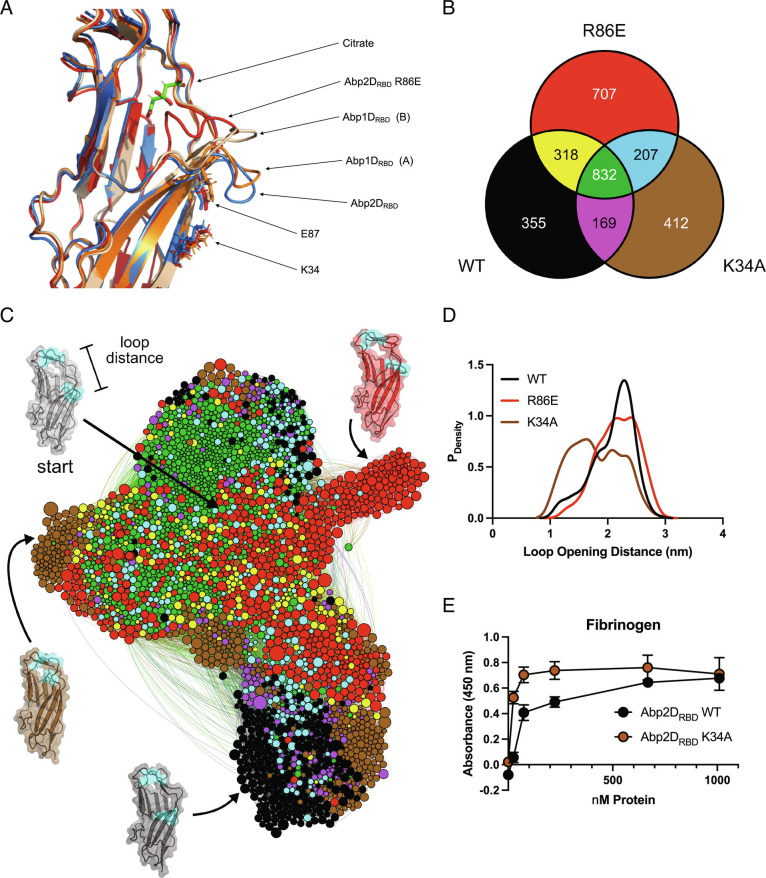
Abp2D_RBD_ pocket dynamics. (*A*) Aligned structures of the two molecules of Abp1D_RBD_ (molecule A—orange and molecule B—wheat), Abp2D_RBD_ (blue), and Abp2D_RBD_ R86E (red), focused on the anterior binding pocket loop. (*B*) Number of unique conformational states in Abp2D_RBD_ WT (black), R86E (red), K34A (brown) and shared between WT/R86E (yellow), WT/K34A (magenta), K34A/R86E (red), and WT/K34A/R86E (green), depicted in *C*. (*C*) Network representation of the shared state-space between variants, as determined by FAST simulations of Abp2D_RBD_ WT, R86E, and K34A. Nodes represent distinct conformational states, with node size proportional to the equilibrium population. Edges represent conditional transition probabilities within 2 ns. Distances between nodes roughly track with their kinetic distance. (*D*) Probability of observing a particular loop opening distance for each variant, determined with a Markov model from the FAST simulations. (*E*) Binding of WT vs. K34A against fibrinogen.

When determining the Abp1D_RBD_ structure, we solved two molecules of this protein in the crystal (asymmetric) unit. By comparing these two molecules, we identified dynamic regions. The structures of the two molecules of Abp1D_RBD_ had a backbone rmsd value of 0.176 Å (Fig. 3*E*), demonstrating that they are highly similar. However, while most of the binding pocket was structurally conserved, one region of substantial variation was a 10 amino acid stretch (R86-S95) encompassing the anterior binding pocket loop. Compared to molecule A Abp1D_RBD_, molecule B’s anterior binding pocket loop was displaced up and into the pocket, moving the α-carbon of S91 by 7.9 Å in the >50 Å long structure. The rmsd value of the backbone of this region was 2.275 Å, indicating that it was substantially distorted from one Abp1D_RBD_ molecule to another (Fig. 5*A*). The anterior loop’s flexibility is significant because this loop can change the shape of the binding pocket, which likely affects protein function.

The different orientations of the Abp1D_RBD_ anterior loop led us to assess possible dynamics of AbpD2_RBD_. We used molecular dynamics (MD) simulations to perform the goal-oriented sampling scheme, FAST (24), on Abp2D_RBD_, focusing specifically on the anterior loop movement seen in the Abp1D_RBD_ crystal structures, which we hypothesize also occur in Abp2D_RBD_. To enhance sampling of this region, we used the distance from the tip of the anterior loop of Abp2D_RBD_ (defined as residues 89 to 92) to the top (residues 104 to 110) of the binding pocket (loop opening distance) to define “open” vs. “closed” and provide a FAST objective. A more “open” pocket has a larger loop opening distance and a “closed” pocket has a smaller loop opening distance. Subsequent analysis showed that equilibrium populations of Abp2D_RBD_ skewed toward a larger pocket opening distance, indicating that the anterior binding pocket loop in Abp2D_RBD_ is flexible but predominantly favors a more open conformation (Fig. 5*D*). The FAST simulation results suggest the dynamic nature of the anterior binding pocket loop is a conserved biophysical feature of this motif.

To assess whether the reduced-binding R86E mutation modulates the anterior loop opening dynamics, we crystallized the Abp2D_RBD_ R86E protein, resulting in a cuboidal crystal, which was solved at 1.9 Å resolution (8 DKA). The electron density of the anterior loop is sparse, starting at position 86, even though the rest of the protein is well-modeled. This indicates that the loop is flexible and oriented in different conformations between different molecules throughout the crystal. However, we were able to model the R86E anterior loop, finding that it is displaced dramatically up and into the binding pocket by 11.6 Å relative to the structure solved for Abp2D_RBD_ WT (Figs. 4*C* and 5*A*). In tandem, we performed FAST simulations using a homology model of Abp2D_RBD_ R86E, generated from the Abp2D_RBD_ WT crystal structure. Consistent with our hypothesis, the R86E mutant ensemble is characterized by a greater diversity of open and closed distances (Fig. 5 *B*–*D*). R86E also exhibits an increase in the total number of heterogeneous conformations, indicating that this anterior binding pocket loop is destabilized by this mutation (Fig. 5*B*). Our findings indicate that the R86E null binding phenotype may in part be caused by the flexibility in the anterior loop in addition to the charge difference resulting from arginine to glutamate side mutation.

To further evaluate residues involved in anterior loop positioning, we assessed a mutant at K34, which is located on the underside of the anterior binding loop motif. The crystal structure of Abp2D_RBD_ shows that K34 forms a salt bridge with E87 (Fig. 5*A*). While these two residues are conserved in the Abp1D_RBD_, they are too far away from each other (>4 Å) (in the crystal structures) to form a salt bridge in both Abp1D_RBD_ molecule A or Abp1D_RBD_ molecule B and the loops are more closed than in the Abp2D_RBD_ structure (especially in molecule B of the Abp1D_RBD_ structure). Therefore, we hypothesized that the salt bridge in Abp2D_RBD_ is restricting the movement of the anterior loop in our simulations. By comparing simulations of an K34A variant of Abp2D_RBD_ to simulations of WT, we found that the K34A mutation releases the anterior binding pocket loop. The K34A FAST structural ensemble favors a smaller loop opening distance and a more closed conformation compared to WT (Fig. 5*D*). Therefore, the K34A salt bridge seen in the Abp2D structure is holding the anterior loop down, keeping the pocket open. Further, from our simulations, we find a high degree of overlap between conformational landscapes of Abp2D_RBD_ WT, R86E, and K34A. From a shared-state space clustering, we find that more than 50% of conformational states are shared, representing 68% of the global population. Additionally, the range of anterior loop opening distances between these variants is found to be very similar, as indicated by the highly overlapping and interconnected state-space (Fig. 5 *C* and *D*). However, their relative populations and rates of transitioning are not similar, evidenced by our projections of individual populations onto anterior loop opening distance (Fig. 5 *C* and *D*). To better assess how these in silico results correspond to protein binding, we compared Abp2D_RBD_ K34A’s fibrinogen binding to Abp2D_RBD_ WT. Abp2D_RBD_ K34A has a higher affinity to fibrinogen as compared to Abp2D_RBD_ WT (Fig. 5*E*). Hence, releasing this anterior loop from its interaction with K34 increases the binding ability of the protein. Thus, by FAST simulation and crystallography, we have defined two residue positions which show the potential to regulate anterior loop dynamics and downstream protein binding with the closed and open conformations resulting in high and low binding, respectively.

### Abp1D- and Abp2D-Binding Tropisms.

**Fig. 6.**
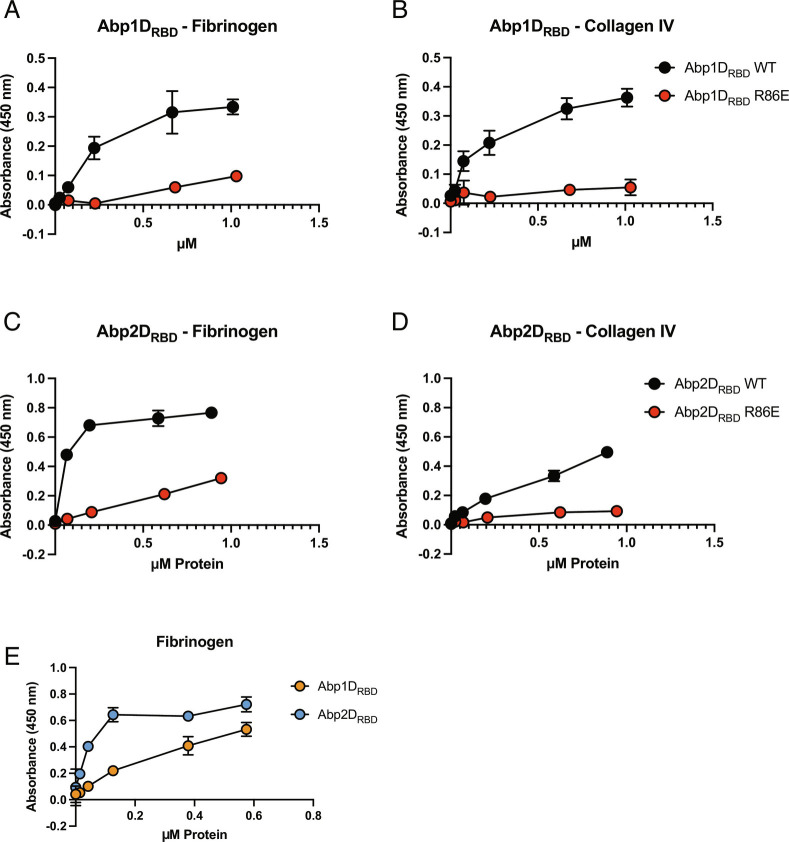
Repertoire of Abp1D_RBD_ and Abp2D_RBD_ binding. ELISA binding to *A* and *C* fibrinogen and *B* and *D* Collagen IV. (*E*) Binding comparing Abp1D_RBD_ and Abp2D_RBD_. Biotinylated WT and R86E protein constructs tested for binding to set of glycoproteins. N = 4 with a background (N = 1) subtracted.

**Fig. 7.**
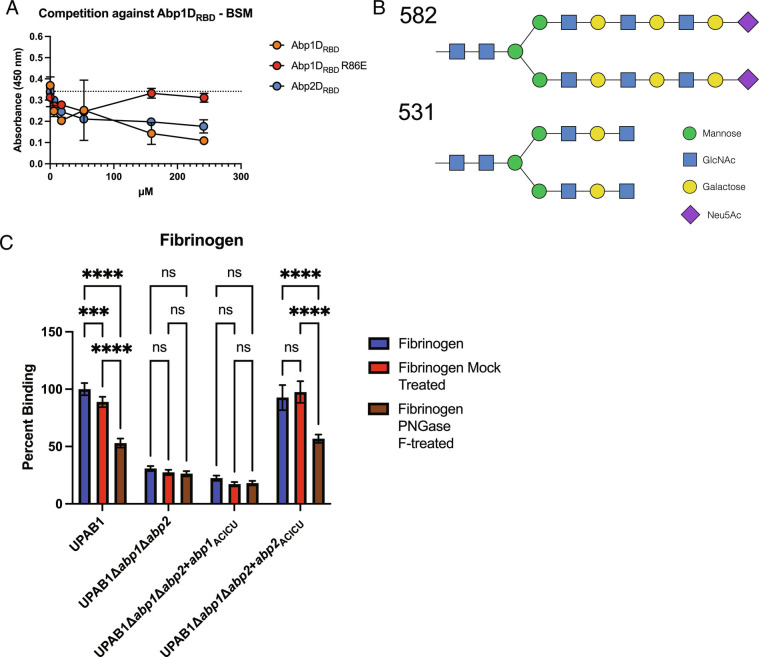
Abp1D_RBD_ and Abp2D_RBD_ receptor binding. (*A*) Competition experiments of labeled Abp1D_RBD_ binding to BSM, competed against unlabeled Abp1D_RBD_, Abp1D_RBD_ R86E, and Abp2D_RBD_. (*B*) Compounds 582 and 531 which represented the highest binders by microarray assay. (*C*) UPAB1 deletion and complementation mutants binding to untreated, mock-treated, and PNGase F-treated fibrinogen. Brown-Forsythe and Welch ANOVA tests. ****P ≤ 0.001, ***P ≤ 0.01, **P ≤ 0.01, *P ≤ 0.05.

We found, through ELISA assays, that Abp1D_RBD_ and Abp2D_RBD_ bind all three tested glycoproteins: fibrinogen, collagen IV, and precipitated urine protein, in addition to the previously tested BSM (Fig. 6 *A*–*D* and *SI Appendix*, Fig. S5 *A*–*D*). R86E mutations in both Abp1D_RBD_ and Abp2D_RBD_ uniformly decrease the binding of the protein construct to each of the tested glycoproteins. Thus, our mutagenesis work demonstrates that Abp1D and Abp2D_ACICU_ share a common mechanism of adhesion to multiple ligands via analogous binding pockets, which share conserved residues critical for binding in Abp2D (R102 and Y141A). Though Abp1D and Abp2D_ACICU_ share residues critical for adherence (R86, R102, and Y141), Abp2D_RBD_ has a higher affinity for fibrinogen than Abp1D_RBD_ (Fig. 6*E*). These results suggest that both Abp1D_RBD_ and Abp2D_RBD_ can independently bind fibrinogen and that the observed differences in residues in the binding pocket between Abp1D_RBD_ and Abp2D_RBD_ affect RBD affinity for its receptor. The majority of the amino acid sequence variation is present on the anterior binding pocket loop and on the right side of the binding pocket. To study whether these proteins were binding to the same or similar receptors, as indicated by the high-throughput screen, we performed experiments wherein a labeled adhesin competed against an unlabeled adhesin in binding to a surface-adherent glycoprotein ligand. Competitive binding experiments indicate that unlabeled Abp2D_RBD_ is able to reduce the signal of labeled Abp1D_RBD_ binding to BSM in a dose-dependent manner (Fig. 7*A*). This demonstrates that Abp1D_RBD_ and Abp2D_RBD_ are binding to the same or an overlapping set of receptors.

To identify the specific molecular receptor for Abp1D and Abp2D, microarray analysis was done via the Center for Functional Glycomics, screening Abp1D_RBD_ and Abp2D_RBD_ for adherence to a panel of glycans, including complex polysaccharides (Table 1). This study found that Abp1D_RBD_ and Abp2D_RBD_ bound to an overlapping subset of glycans, particularly compounds 582 and 531 which exhibited the highest binding for both proteins (Table 1) (Fig. 7*B*). Decorated with additional glycosylations, these compounds contain a base biantennary (NA2) glycan, which consists of two N-acetylglucosamines (GlcNAcs), with a central trimannose moiety, and an GlcNAc and terminal galactose (Gal) on each branch from the trimannose (25, 26). NA2 is a common glycan present in the human host (27, 28). In particular, NA2 is an N-linked glycan on fibrinogen (27). To evaluate this ligand, we treated fibrinogen with PNGase F (N-linked glycosidase) and tested *A. baumannii*’s ability to adhere to the treated fibrinogen. PNGase F treatment of fibrinogen significantly reduced UPAB1 binding to fibrinogen (Fig. 6*C*). Such treatment also reduced the ability of UPAB1Δ*abp1*Δ*abp2* complemented with *abp2* from ACICU to bind fibrinogen (Fig. 6*C*). This indicates that *A. baumannii* can adhere to fibrinogen, at least in part, through the N-linked NA2 glycan with additional glycosylations.

**Table 1.** Highest binding partners for Abp1D_RBD_ and Abp2D_RBD_

Chart ID	Glycan	Abp1D_RBD_		Abp2D_RBD_	
		Average RFU	SD	Average RFU	SD
582	Neu5Aca2-3Galb1-4GlcNAcb1-3Galb1-4GlcNAcb1-3Galb1-4GlcNAcb1-2Mana1-6(Neu5Aca2-3Galb1-4GlcNAcb1-3Galb1-4GlcNAcb1-3Galb1-4GlcNAcb1-2Mana1-3)Manb1-4GlcNAcb1-4GlcNAcb-Sp12	9,786	418	59,054	1,208
531	GlcNAcb1-3Galb1-4GlcNAcb1-2Mana1-6(GlcNAcb1-3Galb1-4GlcNAcb1-2Mana1-3)Manb1-4GlcNAcb1-4GlcNAcb-Sp12	6,977	1,001	59,914	1,963
470	Neu5Aca2-3Galb1-4GlcNAcb1-2Mana-Sp0	6,747	133	53,822	4,116
535	GlcNAcb1-3Galb1-4GlcNAcb1-3Galb1-4GlcNAcb1-2Mana1-6(GlcNAcb1-3Galb1-4GlcNAcb1-3Galb1-4GlcNAcb1-2Mana1-3)Manb1-4GlcNAcb1-4GlcNAcb-Sp12	4,735	602	34,056	4,827
472	Neu5Aca2-6Galb1-4GlcNAcb1-6GalNAca-Sp14	4,714	450	54,517	1,322
285	Galb1-3GlcNAcb1-3Galb1-3GlcNAcb-Sp0	3,392	319	28,674	4,137
439	Fuca1-2Galb1-4(Fuca1-3)GlcNAcb1-2Mana1-6(Fuca1-2Galb1-4(Fuca1-3)GlcNAcb1-4(Fuca1-2Galb1-4(Fuca1-3)GlcNAcb1-2)Mana1-3)Manb1-4GlcNAcb1-4GlcNAcb-Sp12	3,373	55	30,667	511
545	GlcNAcb1-3Galb1-4GlcNAcb1-6(GlcNAcb1-3Galb1-4GlcNAcb1-2)Mana1-6(GlcNAcb1-3Galb1-4GlcNAcb1-2Man a1-3)Manb1-4GlcNAcb1-4GlcNAc-Sp24	3,159	350	39,436	6,368
412	Galb1-4(Fuca1-3)GlcNAcb1-2Mana1-6(Galb1-4(Fuca1-3)GlcNAcb1-2Mana1-3)Manb1-4GlcNAcb1-4(Fuca1-6)GlcNAcb-Sp22	2,932	266	48,357	11,791
356	Fuca1-2Galb1-4(Fuca1-3)GlcNAcb1-2Mana1-6(Fuca1-2Galb1-4(Fuca1-3)GlcNAcb1-2Mana1-3)Manb1-4GlcNAcb1-4GlcNAb-Sp20	2,867	192	58,018	2,343
24	(3S)Galb1-4(Fuca1-3)(6S)Glc-Sp0	2,649	234	42,488	2,652
459	Neu5Aca2-6Galb1-4GlcNAcb1-6(Neu5Aca2-6Galb1-4GlcNAcb1-2)Mana1-6(GlcNAcb1-4)(Neu5Aca2-6Galb1-4GlcNAcb1-4(Neu5Aca2-6Galb1-4GlcNAcb1-2)Mana1-3)Manb1-4GlcNAcb1-4GlcNAcb-Sp21	1,459	1,442	37,002	2,336

Top two molecules contain the base NA2 glycan structure. Data collected from center for functional glycomics microarray. Microarray performed at 50 μg/mL.

## Discussion

Adhesion to host tissues is a key step in pathogenesis. Several *A. baumannii* adhesins have been identified, including Bap, Ata, and InvL (29–32). However, the role of CUP adhesins in *Acinetobacter* uropathogenesis has not been extensively investigated. In this work, we have uncovered the molecular and structural basis for *A. baumannii* CAUTI pathogenesis. This work identifies two widely distributed factors, Abp1D and Abp2D, in *A. baumannii* and provides a mechanistic understanding of their function in *A. baumannii* CAUTI.

A growing body of literature shows that the coating of a catheter by host fibrinogen is a critical step exploited by both Gram-negative and Gram-positive bacteria for the establishment of CAUTI pathogenesis. This was first shown for *Enterococcus* sp, which makes use of the fibrinogen-binding EbpA adhesin located at the tip of the Ebp sortase-associated pilus (6) to colonize the catheter. Methicillin-resistant *Staphylococcus aureus* uses the adhesive clumping factor ClfB for catheter colonization (7). In our current work, we show that MDR *A. baumannii* use the CUP adhesin proteins Abp1D and Abp2D to bind fibrinogen and colonize the catheter. We have demonstrated that each *Acinetobacter* pilus (Abp1 and Abp2) separately contributes to uropathogenesis by mediating adherence to the host factor fibrinogen. We found that Abp1 and Abp2 are present in a high percentage of *A. baumannii* strains*,* and here, we have unveiled their role in device-associated infections. Whether they are important in other types of infections outside of the urinary tract remains to be studied. We hypothesize that this may be the case, as both Abp1D_RBD_ and Abp2D_RBD_ recognized receptors containing the NA2 glycan, which is a common N-linked glycan present on various human glycoproteins, including fibrinogen (25, 27, 28). As an opportunistic pathogen, *A. baumannii* likely benefits from the ability to attach to such a ubiquitous host receptor.

The crystal structures of Abp1D and Abp2D_ACICU_ RBD revealed that they were structurally highly similar, particularly within a shared anterior binding pocket loop motif. Biophysical, biochemical, and structure–function analyses defined residues of Abp1D and Abp2D needed for binding. In addition, we defined residues that affect positioning of the anterior loop structure that controls the opening (low binding) and closing (high binding) of the binding pocket in part via a salt bridge interaction. Interestingly, this loop motif is found in several other γ4 clade RBD, and R86, at the base of Abp1D- and Abp2D-binding pockets, is located in an analogous region to the pockets of other γ4 clade receptor binding domains. We discovered that an R86E mutation attenuates fibrinogen binding. The crystal structure of R86E Abp2D_RBD_ and FAST simulations of R86E and K34A revealed that such mutations destabilize the anterior loop in Abp2D_RBD_, allowing it greater flexibility. For R86E, the electrostatic consequences of the side chain charge inversion, necessary to eliminate binding at this position, could be contributing to the flexibility of the anterior loop motif. K34 forms a salt bridge with E87, which controls the opening (low binding) and closing (high binding) of the pocket by regulating the movement of this anterior loop. Eliminating the electrostatic interaction of K34-E87 via K34A dysregulates the anterior loop by allowing it to flip up. This would form a more closed binding pocket for a ligand and, functionally, results in a higher affinity protein compared to WT.

The regulation of binding due to adhesin conformational equilibriums has been described for the *E. coli* adhesin, FimH, which is located at the tip of the type 1 pilus. FimH is a two-domain CUP adhesin composed of a receptor-binding domain and a pilin domain that connects it to the pilus (similar to Abp1 and Abp2). FimH exists in an equilibrium between relaxed and tense conformations (15). In the relaxed conformation, the binding pocket is formed, allowing tight receptor binding to mannose. However, in the tense conformation, the binding pocket is deformed leading to weak mannose binding (15). Mutants have been made shifting the equilibrium toward either relaxed or tense. Interestingly bacteria carrying either type of mutation display attenuated virulence, suggesting that the equilibrium between conformers is important in pathogenesis (15). Evidence of this phenomenon also exists for the γ4 Type 3 adhesin, MrkD, which was used to solve the Abp2D_RBD_ structure. Two studies demonstrate that mutating T52 in MrkD, which is analogous to K34 in Abp1D and Abp2D, can increase (T52S) or decrease (T52I) the binding of the pilus depending on the substituted residue (23, 33). This MrkD phenotype may be explained by the potential influence of T52 on the dynamic nature of the anterior loop motif, as we report here with K34. Further studies of the dynamic nature and binding properties of CUP adhesins, which are ubiquitous in gram-negative bacteria, including important pathogens that are becoming increasingly antibiotic resistant, will lead to a greater understanding of potential tropisms of common and emerging pathogens and give insight into their virulence mechanisms.

## Methods

### Bacterial Strain Growth.

Bacterial stocks were maintained as glycerol stocks at −80 °C. Strains were streaked out, and the resulting colonies were used for experiments and expression as noted herein. Unless otherwise noted, bacteria were grown in LB or low-salt LB with appropriate antibiotics for selection when needed and, if indicated, at 37 °C under shaking.

### Construction of *A. baumannii* Mutant Strains.

Primers used in this study are listed in *SI Appendix*, Table S2. Mutants in UPAB1 were constructed using standard protocols described previously (34). Briefly, an antibiotic resistance cassette was amplified with 150 bp oligonucleotide primers (Integrated DNA Technologies) with homology to the flanking regions of the targeted gene with an additional 3′ 18 to 25 nucleotides of homology to the FRT site-flanked apramycin resistance cassette from plasmid pKD4 (35). This PCR product was electroporated into competent UPAB1 carrying pAT04, which expresses the RecAB recombinase. Mutants were selected on 30 µg/ml apramycin, and integration of the resistance marker was confirmed by PCR. To remove the apramycin resistance cassette, electrocompetent mutants were transformed with pAT03 plasmid, which expresses the FLP recombinase. All mutant strains were confirmed by PCR and sequencing. Genetic complementation was achieved using a mini-Tn7 system as previously described (36, 37). Briefly, pUCT18-miniTn7-Zeo was amplified and fused together with the operon of interest along with the upstream putative promoter region. The copy of the complemented gene and the zeocin resistance cassette were then introduced into the chromosome using a four-parental conjugation technique. Complementation was confirmed by PCR analysis.

Deletion mutants in ACICU were constructed via a suicide vector containing the sacB marker (pEX18Tc) and sucrose counterselection as previously described (38). Assembly of DNA fragments was performed using the In-Fusion HD EcoDry Cloning Kit (TaKaRa Bio, Mountain View, CA). Briefly, ~1 kb regions upstream and downstream of target genes were amplified from ACICU genomic DNA (gDNA) and fused together linearized pEX18Tc vector. The resulting suicide vector was confirmed by PCR and transformed by electroporation into ACICU. Transformants were selected on tetracycline plates to yield single-crossover mutants. Counterselection was carried out at room temperature on LB plates with 10% sucrose and no NaCl, and colonies were screened by PCR for the deletion.

### Murine CAUTI Model.

Six- to eight-wk-old female C57BL/6 mice were obtained from Charles River Laboratories. Mice were transurethrally implanted with a small piece of silicone tubing (catheter implant) and inoculated as previously described (1). Briefly, mice were anesthetized by inhalation of 4% isoflurane, and a 4- to 5-mm piece of silicone tubing (catheter) was placed in the bladder via transurethral insertion. Bacterial strains were prepared for inoculation after static growth twice overnight at 37 °C by centrifugation at 6,500 rpm for 5 min, washing twice in 1× PBS, and resuspension in PBS to the final inoculum. When indicated, mice were infected immediately following implant placement with ∼2 × 10^8^ CFUs bacteria in 50 μL via transurethral inoculation. At 24 h postinfection (hpi), mice were sacrificed, and kidneys, bladders, and implants were aseptically removed. The bacterial load present in each tissue was determined by homogenizing each organ in PBS and plating serial dilutions on LB agar supplemented with antibiotics when appropriate. All CAUTI studies were performed in accordance with the guidelines of the Committee for Animal Studies at Washington University School of Medicine, and we have complied with all relevant ethical regulations. Mice were housed with a cycle consisting of 12 h of light and dark with access to standard food and water ad libitum.

### Biofilm Assays.

Overnight static cultures were normalized to an OD_600_ of 0.05 in human urine (IRB: 201901098) or human urine with human fibrinogen (150 µg/ml) (Sigma) and inoculated into sterile, round-bottomed, polystyrene 96-well plates (Corning Inc.) with 200 µL final volume per well. Plates were incubated for 24 h under static conditions at 37 °C with humidity to prevent evaporation. After incubation, planktonic cells in the culture were removed and transferred to another 96-well plate and OD_600_ readings were taken using a BioTek microplate reader. Bacteria bound to the wells of the overnight cultures were washed 5 times with water and stained with 300 µL of 0.1% crystal violet solution dissolved in water for 15 min. After staining, wells were washed 5 times with water and left to completely dry overnight. Crystal violet staining was dissolved in 200 µL of 30% acetic acid, and absorbance was measured at 550 nm.

### Cell Adherence Assays.

Flat-bottom microplates (Thermo Fisher) were coated overnight at 4 °C with BSM (10 µg/ml) (Sigma) and human fibrinogen from plasma (150 µg/ml) (Sigma). The plates were blocked for an hour with 1.5% BSA in PBS, followed by washing with PBS (three times for 5 min each). Bacterial strains were grown overnight in LB broth, normalized to an OD_600_ of 0.5, and then washed and resuspended in PBS. A total of 100 µL of bacteria was added to the coated wells and incubated for 2 h at 37 °C, followed by PBS washes to remove the unbound bacteria. Next, bacterial cells were fixed with formalin for 20 min at room temperature, followed by three washes with PBS containing 0.05% Tween 20 (PBS-T). Then, the plates were blocked overnight at 4 °C with 1.5% BSA-PBS, followed by three washes with PBS-T. After the washes, the plates were incubated for 2 h at room temperature with rabbit anti–*Acinetobacter* antisera (1:1,000). Plates were washed with PBS-T, incubated with the Odyssey secondary antibody (goat anti-rabbit IRDye680LT, diluted 1:10,000) for 45 min at room temperature, and washed with PBS-T (three times). The plates were scanned for infrared signals with the Odyssey Imaging System (LI-COR Biosciences).

### Gene Carriage Analysis.

To interrogate for presence of Abp1D and Abp2D, a searchable *k-mer* database of 661 K curated genomes was utilized using the COBS search index (https://github.com/bingmann/cobs, checkout 7c030bb) (20), with a threshold value of 0.80. Phylogenetic analysis was performed using Mega X (39).

### Protein Representations.

Protein alignments were conducted using Clustal Omega, Multiple Sequence Alignment (https://www.ebi.ac.uk/Tools/msa/clustalo/) on default settings (22). Alignments were performed with the mature, non-His tagged primary protein sequence. Protein tertiary structure figures were generated in Pymol (Cartoon and surface representations). Protein volume analyses were performed through the Computed Atlas of Surface Topography of proteins (CASTp) server (http://sts.bioe.uic.edu/castp/index.html?2was) (40). Volume representation figures were generated in this program. Protein topologies figures were generated using Pro-orgami (41). Secondary structural elements were determined using DSSP. Domain decomposition was determined by DDOMAIN. The coordinate file HELIX and SHEET charts contributed to secondary structure assignment. Helices were determined using the distance matrix.

### MD Simulations.

MD simulations were run with Gromacs 2020.1 at 310 K using the AMBER99SB-ILDN force field with explicit TIP3P solvent (42–44). Wild-type simulations were prepared by placing the starting structure (PDB ID: 8DEZ) in a dodecahedron box that extends 1.0 Å beyond the protein in any dimension. Alleles K34A and R86E were prepared similarly, with mutations generated in PyMOL. Each system was then solvated and energy minimized with a steepest descents algorithm until the maximum force fell below 100 kJ/mol/nm using a step size of 0.01 nm and a cutoff distance of 1.2 nm for the neighbor list, Coulomb interactions, and van der Waals interactions. For production runs, all bonds were constrained with the LINCS algorithm and virtual sites were used to allow a 4 fs time step (45, 46). Cutoffs of 1.0 nm were used for the neighbor list, Coulomb interactions, and van der Waals interactions. The Verlet cutoff scheme was used for the neighbor list. The stochastic velocity rescaling (47) thermostat was used to hold the temperature at 310 K. Conformations were stored every 20 ps.

The FAST algorithm (24) was used to enhance conformational sampling of the binding-pocket proximal loop (residues 89 to 92). For each allele, FAST-distance simulations were run for 5 rounds with 10 simulations per round, where each simulation was 50 ns in length (2.5 μs aggregate simulation per system). The FAST-distance ranking function favored restarting simulations from states that minimized pairwise distances between backbone heavy atoms on loops adjacent to the binding pocket (residues 89 to 92 and 104 to 110). Additionally, a similarity penalty was added to the ranking to promote conformational diversity in starting structures, as has been described previously (48).

Markov State models (MSMs) were built from the FAST simulation data using enspara (49). An MSM is a network representation of a free-energy landscape, where nodes are discrete conformational states and directed edges are conditional transition probabilities. A shared-state space between all variants was defined using backbone heavy atoms (atoms C, C_α_, C_β_, N, and O) and clustered with a *k*-centers algorithm based on rmsd to generate 3,000 cluster centers. Following clustering, an MSM was built for each variant by row-normalizing the observed transition counts, at a lag-time of 2 ns, with a small pseudocount as a prior (50).

### PNGase F Treatment.

N-linked oligosaccharides from fibrinogen were enzymatically removed with PNGase F (New England BioLabs) following the manufacturer’s protocol. Briefly, 100 µL of glycoprotein denaturing buffer was added to 1 mL of 1.5 mg/ml fibrinogen in PBS. The mix was incubated for 10 min at 100 C. After 100 µL glycobuffer 2, 100 µL of 10% NP-40, and 3 µL PNGase F were added to the mix and incubated at 37 °C for 2 h. The glycosylation status was checked by gel electrophoresis and Coomassie blue staining of the samples. For protein-binding studies, fibrinogen was adhered to 96-well ELISA plates and then treated or mock-treated with PNGase F generally in line with its described protocol.

### Cloning and Expression of Protein Constructs.

The target receptor-binding domain gene sequences along with their signal sequences were PCR amplified from appropriate *A. baumannii* gDNA preparations, using a 5′ primer containing an EcoRI recognition site and homologous to sequences upstream of the signal sequence and a 3′ primer containing a BamHI recognition site and a 6× His tag. The PCR product was digested with EcoRI and BamHI and ligated into a similarly cut pTRC99a vector and transformed into competent cells. Expression plasmid constructs were confirmed by sequencing. Amino acid substitution constructs were generated via quick-change PCR, with a WT template. Protein was expressed and harvested in a large-scale fermenter format from C600 containing expression plasmids, grown to logarithmic phase, and induced with IPTG for ~1 h. The culture was subsequently harvested, and the periplasm isolated generally as described previously (15). RBD protein constructs and mutants were purified by cobalt affinity chromatography (eluted by imidazole) and cation (S) exchange chromatography (eluted by NaCl). Purified protein was subsequently dialyzed or buffer exchanged into 20 mM MES 5.79 to 5.8 + 50 mM NaCl or appropriate buffer.

### Protein Labeling.

Protein was biotinylated using the EZ NH4 biotinylation reagent and diluted in either H_2_O or DMSO at 100 mM stock. Protein was either dialyzed or buffer exchanged into 1× PBS. Biotinylation reagent was added at a 20 molar fold excess for 2 h at 4 °C under rocking. Biotinylated protein was subsequently dialyzed into the appropriate assay buffer, removing the excess biotin reagent.

### Western Blots.

Cells grown 2 × 24 under static conditions at 37 °C were resuspended in Laemmli buffer to a final OD of 10. Samples were loaded in a 12% polyacrylamide gel for separation. The rabbit anti-Abp1A or anti-Abp2A (9) and mouse anti-RNA polymerase (Biolegend, San Diego, CA) were both used at a concentration of 1:1,000. Secondary IR dye antibodies from Licor were used at 1:10,000. All blots were blocked in TBS blocking buffer (Licor) and visualized with an Odyssey CLx imaging system (LI-COR Biosciences).

### Glycan Array.

Biotinylated proteins in 1× PBS were sent to the Center for Functional Glycomics (Boston, MA, USA). Microarray assays were performed at 5 and 50 µg/mL on the v5.4 microarray.

### Crystallography and Diffraction Data Collection and Solving.

Abp2D_RBD_ from ACICU and Abp1D_RBD_ from UPAB1, each in 20 mM MES 5.8 + 50 mM NaCl, were concentrated to 9.218 and 4.690 mg/mL, respectively. In a 1:1 drop, Abp2D_RBD_ from ACICU was crystallized over 0.1 M LiSO4, 0.1 M Na citrate, and 15% ethanol. Crystals took approximately 1 wk to appear and formed long, narrow rods. Crystals were looped and cryoprotected in 0.1 M lithium sulfate, 0.1 M Na citrate pH 5.5, 15% ethanol, and 20% glycerol. In a 1:1 drop, Abp1D_RBD_ crystallized over 0.8 M AmSO_4_ and 0.025 M citric acid pH 3.56. Crystals took approximately 1 wk to appear and formed trapezoidal-prism-like shapes. Crystals were looped and cryoprotected in 0.8 AmSO4, 0.025 M citric acid, and 20% glycerol. Abp2D_RBD_ R86E was crystallized at 8.84 mg/mL in 20 mM MES 5.79 + 50 mM NaCl over a well solution of 0.2 M AmSO_4_, 20% Isopropanol, 16% PEG 4000, and 0.1 M HEPES 7.5. Crystals were looped and cryoprotected in ~0.18 M AmSO_4_, ~18% isopropanol, ~0.9 M HEPES 7.5, ~15% PEG 4000, and ~18% glycerol. Data were collected at ALS 4.2.2 macromolecular crystallography beamline. The dataset was solved by molecular replacement and refinement performed in Phenix, using MrkD (3U4K) for Abp2D, Abp2D (8DEZ) for R86E, and a trimmed model of Abp2D (8DEZ) for Abp1D.

### ELISA Binding Assays.

Ligand glycoprotein was added to either HBX4 (Immulon) or Microlon600 (Greiner) plates and allowed to dry overnight onto the well bottom. Commerically available human fibrinogen and human (placental) Collagen IV were used. The wells were then blocked for ≥ 4 h to overnight at 4 °C with a blocking buffer of 1× PBS + 2% BSA + 0.05% Tween-20. Protein was added and allowed to incubate with adhered ligand overnight at 4 °C diluted in a blocking buffer. Wells were then washed 3× with wash buffer of 1× PBS + 0.05% Tween-20. Streptavidin-HRP was added at 1:1,000 dilution in a blocking buffer and incubated for ~1 h at 4 °C. Wells were washed 2× with wash buffer and then 1× with 1× PBS. Wells were developed with 1:1 tetramethylbenzidine development reagent and quenched with ~1 M H_2_SO_4_. Wells were read at 450 nm.

### BLI.

BLI was conducted on an Octet Red 96-well unit. Streptavidin pins were soaked for at least 10 min in a blocking buffer of 1× PBS + 2% BSA + 0.05% Tween-20. The assay was conducted with all analytes and ligands diluted in a blocking buffer. Abp2D_RBD_ protein constructs were used at 2.07 µM. The assay was conducted by the following: baseline in blocking buffer was collected for 60 s (seconds), loading of the biotinylated BSM (in 1× PBS) for 300 s, baseline in blocking buffer collected for 60 s, association with protein collected for 300 s, and dissociation in blocking buffer for 300 s. Data were collected on the Octet Data Acquisition 9 software. Baseline data were subtracted from the protein sample curves in GraphPad Prism to generate the data.

### CD.

Purified samples (at 50 µg/mL) of the RBD of Abp2D WT, R86E, and R86A were prepared in 20 mM phosphate pH 7. Then, 150 µL of sample in a Starna Cell 16.100F-Q-10/Z15 or similar cuvette was used. Three sample curves and three buffer curves were collected for each sample. The buffer curves were subtracted from the sample data. The data were collected on a Chirascan spectrometer unit and data were analyzed using Prism GraphPad software.

### SEC.

Purified samples of Abp2D_RBD_ WT, R86E, and R86A were prepared in 20 mM MES 5.79 + 50 mM NaCl. The Superdex 200 sizing column was brought to equilibrium with 20 mM MES 5.79 + 50 mM NaCl. The samples were manually injected, and the flow rate was 0.5 mL/min. All samples were conducted in sequence on the same day.

### Isolation of Urine Protein.

Urine protein was isolated as described in Conover et al. 2016 (51) and collected under IRB 201207143. Clean catch urine, with the first few mLs discarded, from a 28-y-old healthy male was spun down to remove aggregates and cellular debris. The soluble protein was precipitated with 40% AmSO_4_ overnight at 4 °C, then spun down to pellet the precipitated protein. That protein was resuspended in ~30 mL of 1× PBS 7.4 and dialyzed against ~500 mL of 1× PBS 7.4. The product was then spun down again to remove the remaining undissolved precipitate and passed through a 0.22-µm filter to preserve the product.

## Supplementary Material

Appendix 01 (PDF)

## Data Availability

X-ray structure data have been deposited in the Protein Data Bank (PDB) (8DEZ, 8DF0, 8DKA). All study data are included in the article and/or *SI Appendix*.
