# Supplementary material for: Structure–function correlates of fibrinogen binding by Acinetobacter adhesins critical in catheter-associated urinary tract infections
Source: Proc Natl Acad Sci U S A. 2023 Jan 18;120(4):e2212694120. doi: 10.1073/pnas.2212694120 (PMC9942807; doi:10.1073/pnas.2212694120)
Supplement: Supplementary file 1 — Appendix 01 (PDF) [file pnas.2212694120.sapp.pdf]

PNAS

Supporting Information for

Structure-function correlates of fibrinogen binding by *Acinetobacter* adhesins critical in catheter associated urinary tract infections

Authors: Kevin O. Tamadonfar[1,2]*, Gisela Di Venanzio[1]*, Jerome S. Pinkner[1,2], Karen W. Dodson[1,2], Vasilios Kalas[1,2], Maxwell I. Zimmerman[3], Jesus Bazan Villicana[1,2], Gregory R. Bowman[4], Mario F. Feldman[1], and Scott J. Hultgren[1,2]

Affiliations:

[1]Department of Molecular Microbiology, Washington University School of Medicine, St Louis, MO, USA.

[2]Center for Women's Infectious Disease Research, Washington University School of Medicine, St Louis, MO, USA

[3]Department of Pathology and Immunology, Washington University School of Medicine, St. Louis, MO 63110, USA

[4]Department of Biochemistry & Molecular Biophysics, Department of Biomedical Engineering, and Center for Biological Systems Engineering, Washington University School of Medicine, St. Louis, Missouri 63110, United States

*Co-first authors

Paste Scott J. Hultgren

Email: hultgren@wustl.edu

This PDF file includes:

Figures S1 to S5

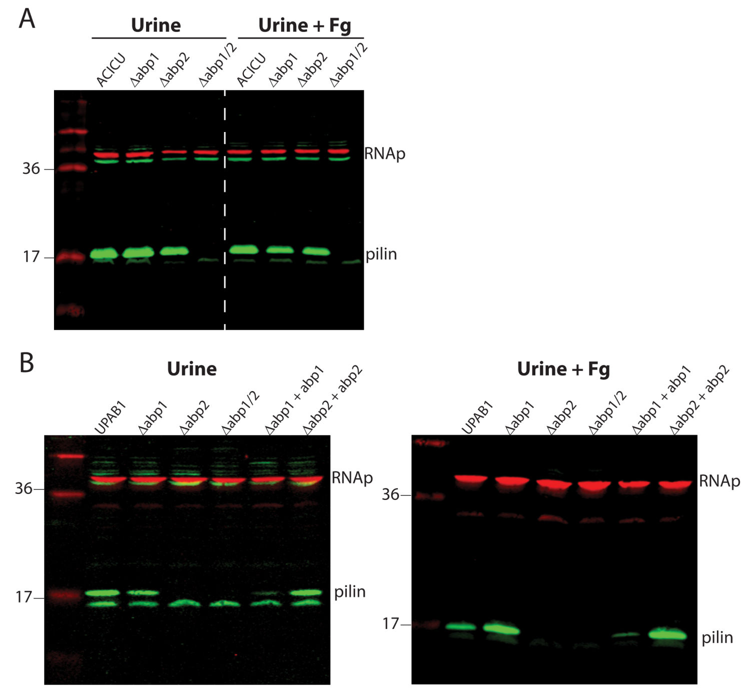

Fig S1: UPAB1 and ACICU expression of Abp1 and Abp2 pili. A) Western blot against the pilin domains of Abp1 and Abp2 pili for WT and mutants of A) ACICU in urine and urine with fibrinogen and B) UPAB1 in urine and urine with fibrinogen.

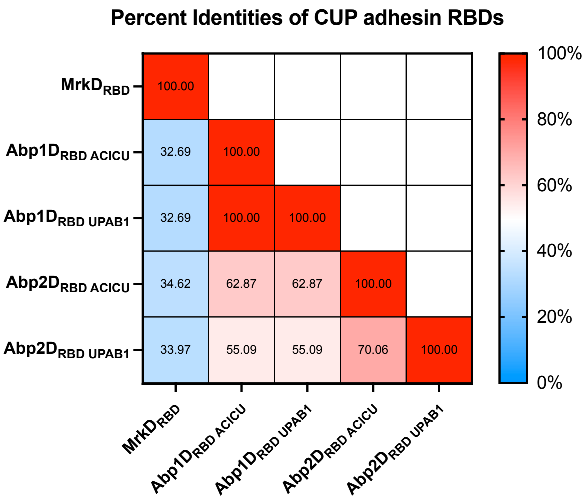

Fig S2: Primary amino acid sequence of various receptor binding domain alleles. Percent identity determined by Clustal Omega from sequences aligned in Figure 3A. MrkD sequence from PDB: 3U4K.

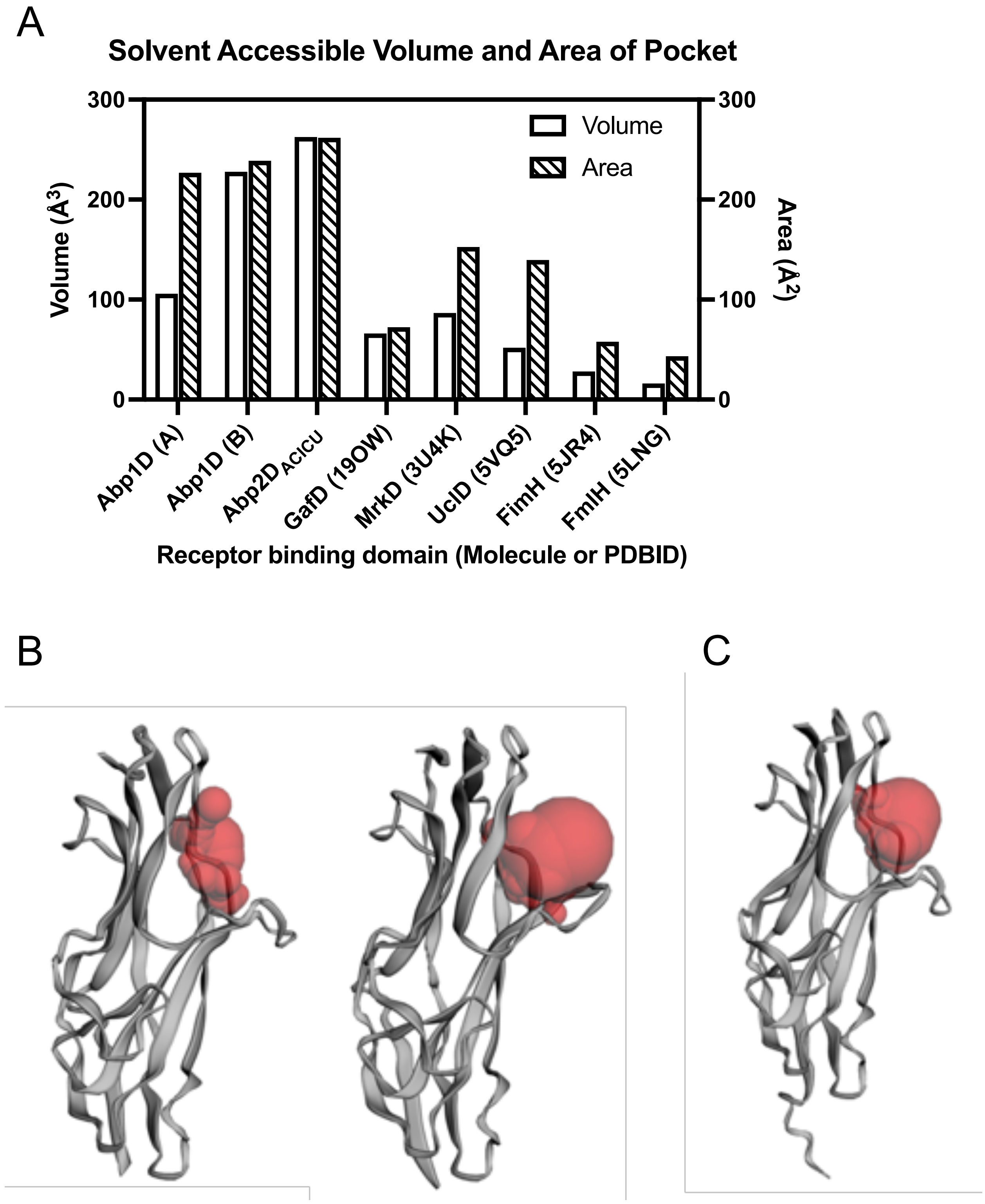

Fig S3: Binding Pocket Volume of γ4 and γ1 CUP adhesin lectin/receptor binding domains. *In silico* calculation by CASTp. A) Quantification of solvent accessible volume and surface area. Visual representation of binding pocket volume in Red of B) two molecules of $Abp1D_{RBD}$ (Molecules A and B from left to right) and C) $Abp2D_{RBD}$.

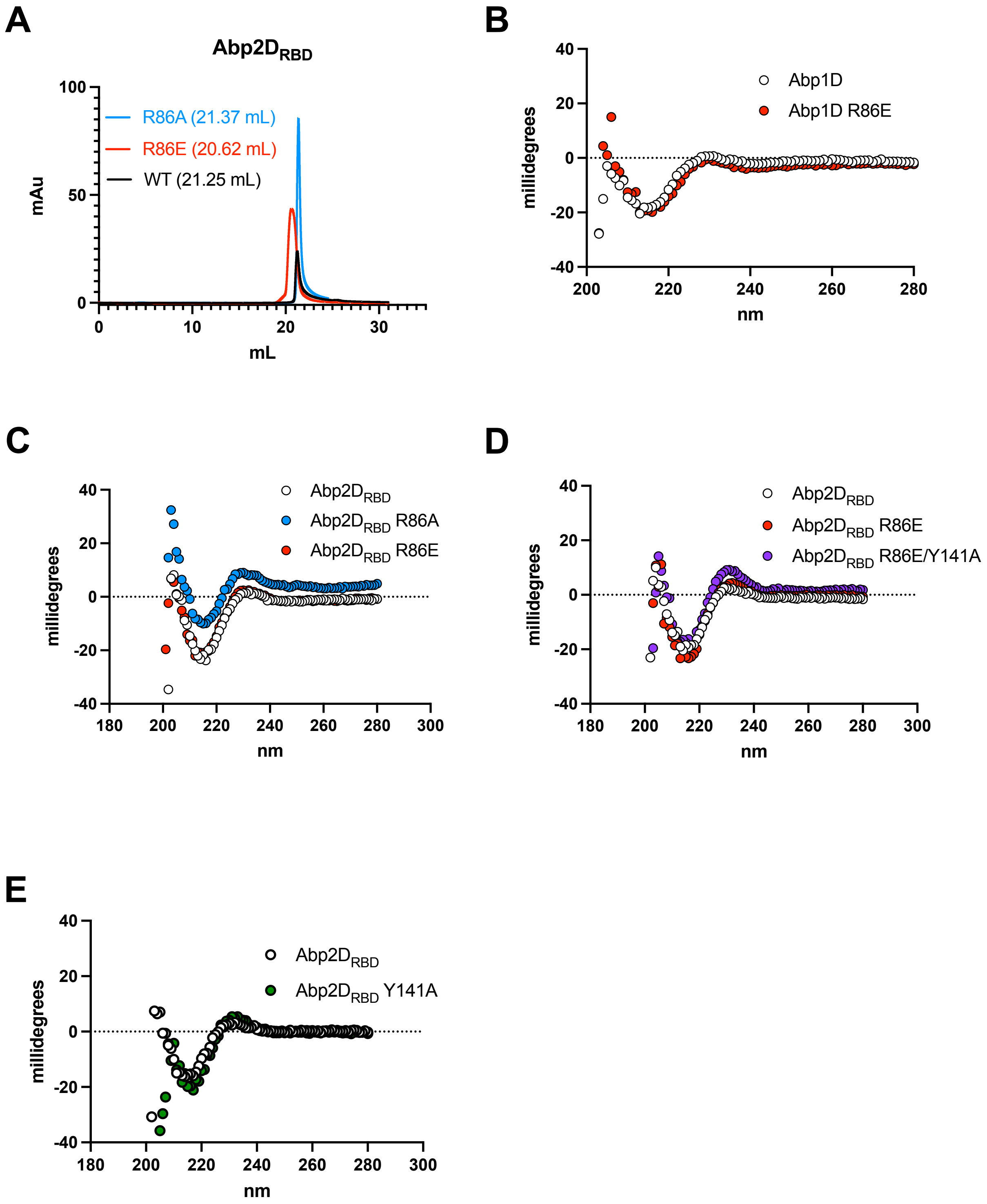

Fig S4: Mutants of the Abp1D and Abp2D RBDs are appropriately folded. A) Size exclusion chromatography profiles of Abp2D$_{RBD}$ WT and R86 mutants. Proteins suspended 20 mM MES 5.79 + 50 mM NaCl. Assayed on Superdex 200 column. In parentheses, elution volume of respective peaks. B-E) Circular dichroism spectra of Abp1D and Abp2D$_{ACICU}$ RBD mutants in 20 mM Phosphate 7.00.

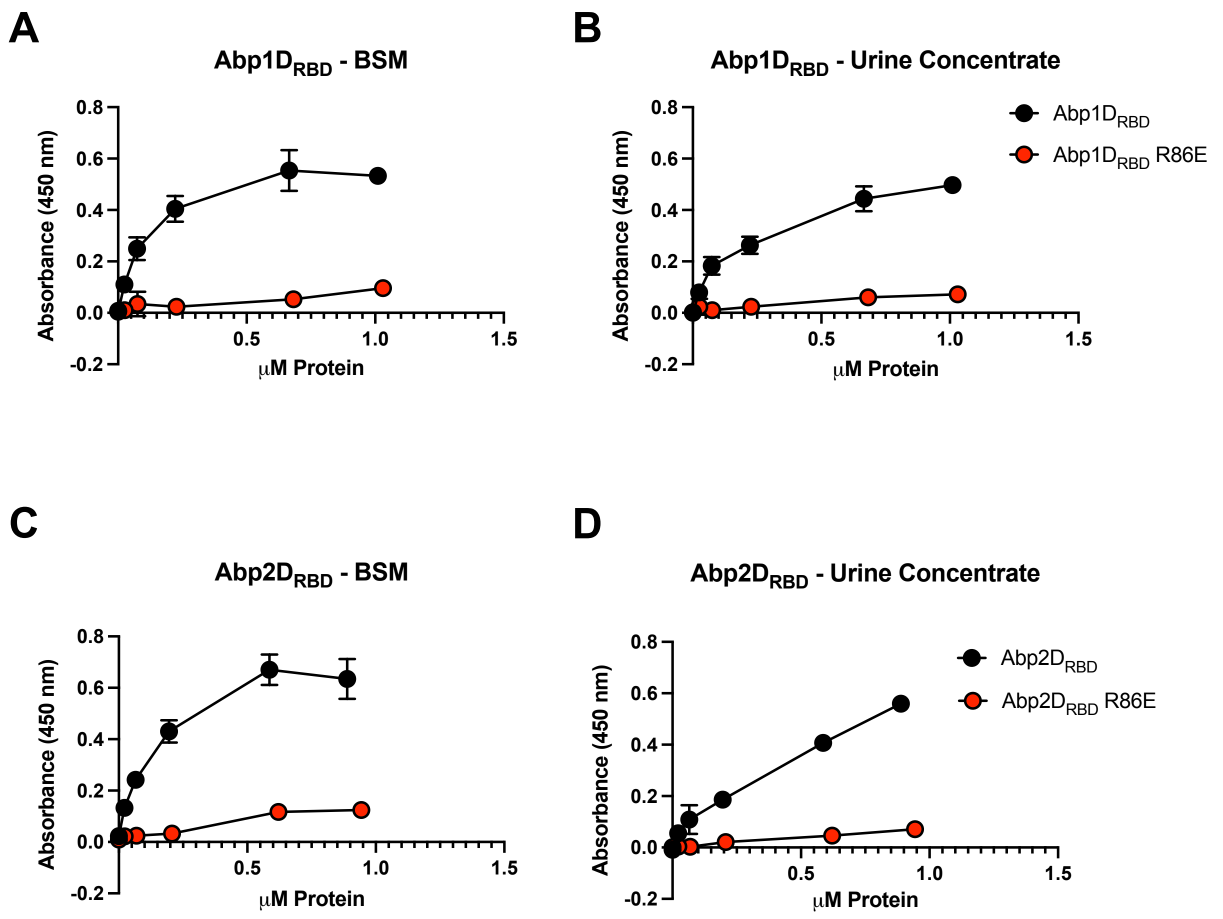

Fig S5: Repertoire of RBD binding. Abp2D$_{RBD}$ binding to A) bovine submaxillary mucin and B) urine concentrate and Abp1D$_{RBD}$ binding to C) bovine submaxillary mucin and D) urine concentrate. Biotinylated Abp2D$_{RBD}$ and Abp2D$_{RBD}$ R86E tested for binding to set of glycoproteins by ELISA. n=4 with a background (n=1) subtracted.

Table S1: Refinement Statistics.

	Abp1D RBD	Abp2D RBD	Abp2D R86E RBD
Resolution range	44.51 - 2.1 (2.175 - 2.1)	47.53 - 1.29 (1.336 - 1.29)	43.8 - 1.9 (1.968 - 1.9)
Space group	P 42 2 2	P 41 21 2	P 21 21 21
Unit cell	91.97 91.97 176.915 90 90 90	51.176 51.176 128.236 90 90 90	36.438 51.847 81.828 90 90 90
Total reflections	629264 (61626)	505499 (19435)	89401 (8836)
Unique reflections	45137 (4370)	42203 (2806)	12772 (1230)
Multiplicity	13.9 (14.0)	12.0 (6.0)	7.0 (7.2)
Completeness (%)	99.76 (99.36)	94.96 (65.35)	99.95 (99.92)
Mean I/sigma(I)	11.90 (0.97)	15.92 (0.63)	15.46 (3.17)
Wilson B-factor	33.97	10.38	22.32
R-merge	0.2715 (2.938)	0.1305 (2.07)	0.1146 (0.8869)
R-meas	0.2818 (3.049)	0.1364 (2.266)	0.124 (0.9574)
R-pim	0.07507 (0.8113)	0.03882 (0.8954)	0.04693 (0.3571)
CC1/2	0.996 (0.434)	0.999 (0.41)	0.998 (0.804)
CC*	0.999 (0.778)	1 (0.763)	0.999 (0.944)
Reflections used in refinement	45041 (4370)	41657 (2804)	12768 (1230)
Reflections used for R-free	2257 (212)	2041 (141)	581 (61)
R-work	0.2258 (0.2972)	0.2028 (0.3137)	0.2117 (0.2518)
R-free	0.2394 (0.3031)	0.2073 (0.3522)	0.2403 (0.2545)
CC(work)	0.945 (0.716)	0.952 (0.710)	0.950 (0.859)
CC(free)	0.936 (0.791)	0.958 (0.616)	0.927 (0.859)
Number of non-hydrogen atoms	2575	1507	1282
macromolecules	2524	1372	1257
ligands	51	31	
solvent	335	104	25
Protein residues	0.01	175	165

RMS(bonds)	1.35	0.008	0.009
RMS(angles)	99.09	1.27	1.16
Ramachandran favored (%)	0.91	97.66	98.76
Ramachandran allowed (%)	0	2.34	1.24
Ramachandran outliers (%)	2.1	0	0
Rotamer outliers (%)	1.59	1.95	0
Clashscore	38.82	0	4.03
Average B-factor	38.93	13.71	28.45
macromolecules	33.58	13.11	28.55
ligands	1	22.58	
solvent		19.03	23.62
Number of TLS groups		1	1

Table S2: Primer list

**Abp1 DS F FRT**	**ATAATGTGTATGATCATTTGACTTTTTTAGTATTAATTAAAAATTATTCATTCTAT AAAGCCTACTACTTACTACTAAGATCTAGTGTTTAGAAGAAATA**
**Abp1 US R FRT**	AAAATAATTTCATGATGTTTTACCATATGGTATAAATTAAAATAAAAACCGCATTT ATCAATAAAAAATAACCAATCAAAATACATAAAAATATAATGTT
**Abp1 out F**	GCAAATTGTGATCTATTTCCC
**Abp1 ut R**	CCAAACTCCAGATGATCTTTTATTG
**Abp2 US R Frt**	TTTGTTTAAAAAGTGTTAGCATTGTTACATTACTTTTCAAGATGTCAAACACTGGA AAAGTAAGGAATTAAACTTTTATCCAGACTTTTGAGAAAACACT
**Abp2 DS F frt**	ATATTCCTATCGAGTTAAGTATGAAATTTTTAGAAAGTAAAAGTTATTATTAATAT TTAATTATTTGAAAATTATTAATTTTATTAACTATTGAATATGA
**Abp2 out F**	ATGTGACTTTTTGGCTAGCC
**Abp2 out R**	TCTTATCTCTATGATTCCTTTCTTC
**Abp1prom inf Fw**	CTCACTAGTGGATCCCAAACTCCAGATGATCTTTTATTG
**Abp1comp inf Rv**	ACCGGGCCCAAGCTTCTAATCATATTGGATCGTTACCG
**Abp2 prom Fw**	CTCACTAGTGGATCCGAAGCTGGCAAAAGAGTG
**Abp2 comp rv**	ACCGGGCCCAAGCTTATTCCTATCGAGTTAAGTATG
**BamHI pEX18 Fw**	GGATCCCCGGGTACC
**HindIII pEX18 Rv**	AAGCTTGGCACTGGC
**US Fw Abp1_ACICU**	GCCAGTGCCAAGCTTGAAGAATTGCCCAGTTAGAAG
**US Rv Abp 1_ACICU**	ATTGAATATTTTGGGCATTTCTTATAAACGCTAGAGC
**DS Fw Abp 1_ACICU**	CCCAAAATATTCAATAAATATCGC

**DS Rv Abp 1_ACICU**	GGTACCCGGGGATCCTTAGGTGAAAGCCGTGAAG
**US Fw Abp 2_ACICU**	GCCAGTGCCAAGCTTTGCTACAGATTTCTTAAATCC
**US Rv Abp 2_ACICU**	TCATATTCAATAGTTTTTAAACC
**DS Fw Abp 2_ACICU**	AACTATTGAATATGATATGTGACCCGTATCTCAC
**DS Rv Abp 2_ACICU**	GGTACCCGGGGATCCATCAGTAAAAGCCGATACTC
